# Pollen Morphology of Some Species from Genus *Nitraria*

**DOI:** 10.3390/plants11182359

**Published:** 2022-09-09

**Authors:** Maria Tomoshevich, Evgeny Banaev, Sofia Khozyaykina, Anna Erst

**Affiliations:** Central Siberian Botanical Garden, Siberian Branch of Russian Academy of Sciences, Zolotodolinskaya Str. 101, 630090 Novosibirsk, Russia

**Keywords:** *Nitraria sibirica*, *Nitraria schoberi*, *Nitraria komarovii*, *Nitraria pamirica*, *Nitraria tangutorum*, *Nitraria praevisa*, pollen structure, light microscopy, scanning electron microscopy

## Abstract

An analysis of pollen grains (in *Nitraria sibirica* Pall., *N. schoberi* L., *N. komarovii* Iljin & Lava ex Bobrov, and *N. pamirica* L. Vassil.) was performed on natural material collected in Russia, Kazakhstan, and Tajikistan. Herbarium specimens from the collection at Komarov Botanical Institute, Russian Academy of Sciences (*N. tangutorum* Bobrov and *N. praevisa* Bobrov) were examined, too. Pollen grains of two species—*N. pamirica* and *N. praevisa*—were studied for the first time. *N. tangutorum* and *N. praevisa* were found to have the perprolate pollen shape, whereas *N. pamirica* was found to have the subprolate shape. An intraspecific differentiation of *N. sibirica* was noted. Populations of *N. sibirica* (Taskarasu, Karatal, and Basshi) possess pollen grains of the subprolate or prolate shape, striate and perforate exine ornamentation, and a longer equatorial axis and a shorter polar axis than other specimens of *N. sibirica*. *N. schoberi* in all populations had anomalous shapes of some pollen grains. Overall, we demonstrated that the length ratio of the polar axis to the equatorial axis, characteristics of pollen in polar view, colpus morphology, and surface ornamentation of pollen grains in the genus *Nitraria* are of great taxonomic importance for the identification of species.

## 1. Introduction

The genus *Nitraria* L. (Nitrariaceae) includes more than 10 species and occurs in the steppe, semidesert, and desert regions of Asia, North Africa, Southeastern Europe (Romania), and Australia [1,2,3,4,5,6,7,8].

Despite the small taxonomic size of the genus and more than a century of research history [9,10,11,12,13,14,15], researchers do not have a clear idea about the number of *Nitraria* species, their genesis, and the systematics of the genus. The main reason is the weak isolation of species according to macromorphological characters, since most of species differ only in the metric characters of leaves and fruits [1,2,16,17,18].

Modern methods for the analysis of *Nitraria* species also cannot elucidate their genesis. In particular, with the help of assays of inter simple sequence repeats (ISSR) markers and sequencing of the internal transcribed spacer (ITS) region and of chloroplast DNA spacers in Inner Mongolia, researchers have identified transitional forms between *N. tangutorum* and *N. roborowskii* as spontaneous hybrids [19]. The existing phylogenetic and biogeographic analyses of the genus by means of DNA sequencing of six regions in the chloroplast genome and of the ITS region [20] are based mainly on specimens from the collection at the Turpan Botanical Garden (Xinjiang Province, China), not from natural habitats. Type specimens have not been studied by those authors, and information about nucleotide variation of the DNA fragments has not been provided.

Therefore, the use of additional sources of information, such as micromorphological characters, may help taxonomists to better delineate species boundaries within the genus. Palynomorphological data are thought to be some of the important parameters for the identification of various problematic taxa. Polarity, shape, size, aperture structure, symmetry, and ornamentation of pollen grains are highly conserved traits and are often utilized to solve complex problems of plant taxonomy [21,22,23,24,25,26].

So, to clarify the independence of the Nitrariaceae family, Agababyan and Tumanyan [27] have used structural features of pollen grains. They provided palynological data on *N. tangutorum*, *N. sibirica*, *N. schoberi*, *N. komarovii*, *N. sphaerocarpa* Maxim., and *N. retusa* (Forsk.) Aschers., and according to this evidence, these species differ in the size of pollen grains, in the structure of furrows and pores, in the thickness of individual layers of the sporoderm, and in the nature of sculptural elements of the exine. Woutersen et al. [28] have employed morphological parameters of pollen grains in research into *Nitraria* phylogeny. They investigated *N. tangutorum*, *N. sibirica*, *N. schoberi*, *N. komarovii*, *N. sphaerocarpa*, *N. retusa*, and *N. billardierei* DC.

Here, we examined the morphology of pollen grains of six species of *Nitraria*, and for two species, such information is reported for the first time. It is noteworthy that the study was conducted on original mass material from natural populations with an assessment of trait variation. This research was designed and conducted with the aim (1) to evaluate the taxonomic significance of pollen features of *Nitraria* taxa and (2) to enlighten the morphology of pollen grains, pollen types using light (LM) and scanning electron microscopy (SEM). We note that in the light of resolution issues of current molecular data, the study of *Nitraria* pollen can assist in the identification of problematic species, which should be covered in any future molecular assessment.

## 2. Results

**Figure 1:**
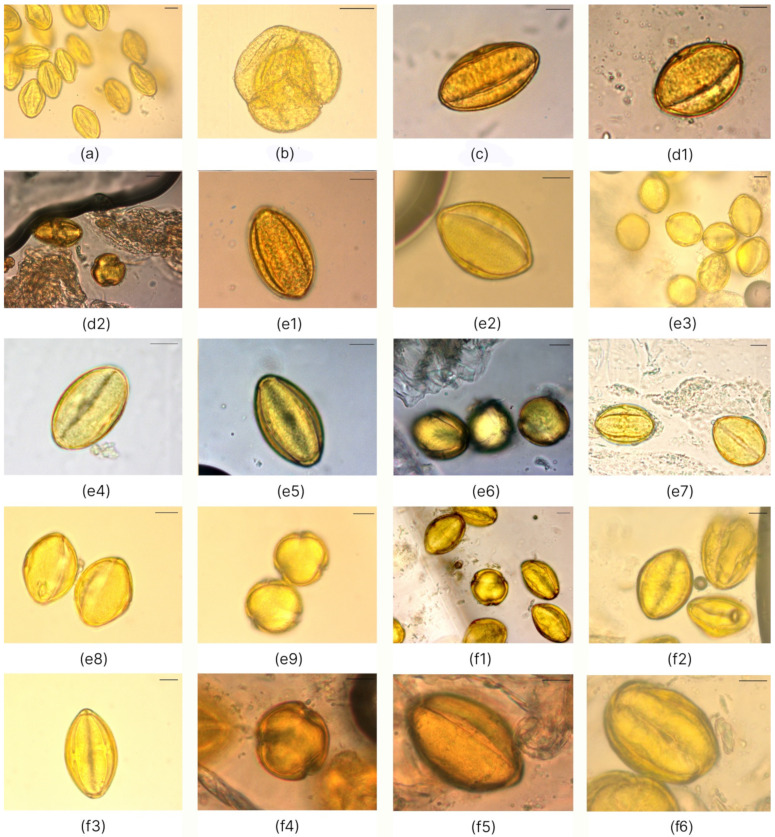
LM micrographs of *Nitraria* pollen: (**a**) equatorial view for *N. komarovii*; (**b**) polar view for *N. pamirica*; (**c**) equatorial view in the mesocolpus area of *N. tangutorum*; (**d1**) equatorial view in the mesocolpus area of *N. praevisa*; (**d2**) semipolar view for *N. praevisa*; (**e1**–**e9**) *N. sibirica* specimens from (**e1**) Balansor, (**e2**) Balhash, (**e3**) Basshi, (**e4**) Dauria, (**e5,e6**) Kosh-Agach, (**e7**) Shelek, and (**e8**,**e9**) Kurti; (**f1**–**f5**) *N. schoberi* specimens from (**f1**) Balhash, (**f2**) Karatal, (**f3**) Malinovoe, (**f4**,**f5**) Sariozek, and (**f6**) Taskarasu. Bar: 10 μm.

**Figure 2:**
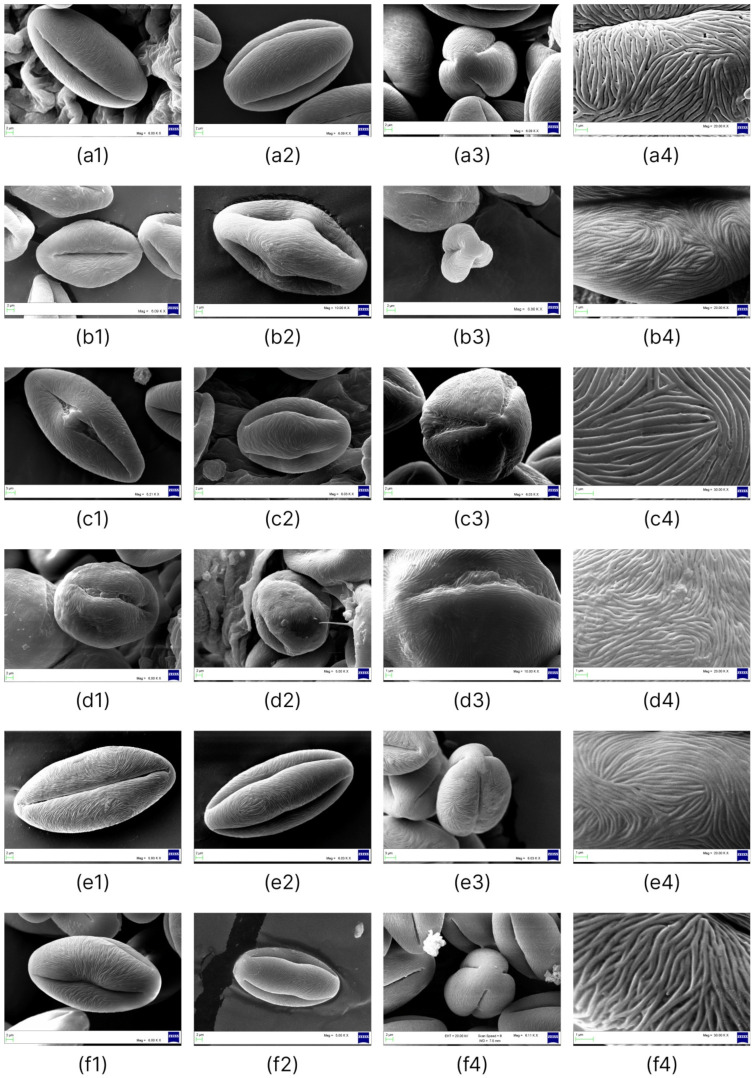
SEM micrographs of *Nitraria* pollen: *N. sibirica*: (**a1**) equatorial view showing a colpus, (**a2**) equatorial view in the mesocolpus area, (**a3**) polar view, and (**a4**) details of the exine surface; *N*. *komarovi*: (**b1**) equatorial view showing a colpus, (**b2**) equatorial view in the mesocolpus area, (**b3**) polar view, and (**b4**) details of the exine surface; *N*. *shoberi:* (**c1**) equatorial view showing a colpus and porus, (**c2**) equatorial view in the mesocolpus area, (**c3**) polar view, and (**c4**) details of the exine surface; *N*. *pamirica:* (**d1**) equatorial view showing a colpus, (**d2**) equatorial view in the mesocolpus area, (**d3**) equatorial view showing a colpus and porus, and (**d4**) details of the exine surface; *N. tangutorum*: (**e1**) equatorial view showing a colpus**,** (**e2**) equatorial view in the mesocolpus area, (**e3**) semipolar view, and (**e4**) details of the exine surface; *N. praevisa*: (**f1**) equatorial view showing a colpus, (**f2**) equatorial view in the mesocolpus area,, (**f3**) polar view, and (**f4**) details of the exine surface.

Detailed characteristics of pollen grains for the six species of *Nitraria* are provided in Table 1 and Figure 1 and Figure 2. The description of pollen traits for each *Nitraria* species is given below.

### 2.1. Morphological Characteristics of the Genus Nitraria

According to Erdtman’s [29] pollen size classification, pollen grains of the *Nitraria* species under study are medium-sized (25–50 μm), and only in *N. schoberi* specimens were there pollen grains up to 50.59 μm (classified as large). Pollen is monad, isopolar, and radially symmetric. The pollen shape is circular, pseudo-hexagonal, hexagonal, or triangular convex in polar view and subprolate to perprolate in equatorial view. The aperture is tricolporate, and colpi are long and narrow, usually constricted at the equator and have costae colpi and a fastigium. The endoapertures are formed by lalongate pores, which are elliptical to rhomboidal in shape. The exine is tectate, with the nexine thicker than the sexine; the surface is striate to striate–perforate.

#### 2.1.1. Morphological Characteristics of *N. sibirica*

Pollen grains of *N. sibirica* are prolate in equatorial view and triangular convex or pseudo-hexagonal, circular in polar view and monad, isopolar, and radially symmetric (Figure 1(e1–e9) and Figure 2(a1–a3)), size: length: 27.60–44.72 μm; width: 13.80–24.71 μm, with an average ratio of the polar axis to equatorial axis (P/E) of 1.70–1.97 (Table 1).

Some specimens of *N. sibirica* have pollen grains that are subprolate or prolate in equatorial view and circular in polar view and have a length of 28.71–37.29 μm, width of 21.10–27.08 μm, with an average P/E ratio of 1.10–1.53.

Aperture: tricolporate; ectoaperture: colpus, almost as long as the polar axis, open (4/5 of polar axis), straight, narrow, occasionally constricted at equator with ends acute; polar area asymmetric. Margin observed in polar view, costae colpi and fastigium conspicuous in equatorial view. Endoaperture: porus, conspicuous, lalongate, elliptic to rhomboidal in shape. Exine: tectate; exine slightly thicker in polar areas in relation to equatorial region; nexine thicker than sexine. Exine ornamentation is striate, no perforations observed. Striae relatively loosely packed and short, running fairly parallel to the polar axis near the colpus and in the mesocolpus area (Figure 2(a4)).

#### 2.1.2. Morphological Characteristics of *N. komarovii*

Pollen grains of *N. komarovii* are prolate in equatorial view and triangular convex to pseudohexagonal in polar view, and they are monad, isopolar, and radially symmetric (Figure 1a and Figure 2(b1–b3)), medium in size: length: 27.15 (23.38–29.50) μm; width: 16.42 (14.23–18.91) μm, with an average P/E ratio of 1.66 (Table 1).

Aperture: tricolporate; ectoaperture: colpus, almost as long as the polar axis, open (2/3 of polar axis), straight, narrow, occasionally constricted at equator with ends acute; polar area asymmetric. Margin observed in polar view, costae colpi and fastigium conspicuous in equatorial view. Endoaperture: porus, conspicuous, lalongate, elliptic to rhomboidal in shape. Exine: tectate; exine slightly thicker in polar areas in relation to equatorial region; nexine thicker than sexine. Exine ornamentation is striate, no perforations observed (Figure 2(b4)). Striae relatively tight and short running parallel to the polar axis near the colpus while running slightly counterclockwise in the mesocolpus area, eventually forming a spiral “cluster” in one area close to a pole or running almost parallel.

#### 2.1.3. Morphological Characteristics of *N. schoberi*

Pollen grains of *N. schoberi* are prolate in equatorial view and triangular convex to hexagonal in polar view, monad, isopolar, radially symmetric (Figure 1(f1–f5) and Figure 2(c1–c3)), medium in size: length: 32.59–50.59 μm; width: 14.23–35.89 μm, with an average P/E ratio of 1.57–1.70 (Table 1).

Aperture: tricolporate. Ectoaperture: colpus, almost as long as the polar axis, open (4/5 of polar axis), straight, narrow, occasionally constricted at equator with ends acute; polar area asymmetric. Margin observed in polar view, costae colpi and fastigium conspicuous in equatorial view. Endoaperture: porus, conspicuous, lalongate, elliptic to rhomboidal in shape. Exine: tectate; exine slightly thicker in polar areas in relation to equatorial region; nexine thicker than sexine. Exine ornamentation is striate, no perforations observed. Striae relatively loosely packed and short, running fairly parallel to the polar axis near the colpus and in the mesocolpus area (Figure 2(c4)).

#### 2.1.4. Morphological Characteristics of *N. pamirica*

Pollen grains of *N.* *pamirica* are subprolate in equatorial view and circular to hexagonal in polar view, and monad, isopolar, and radially symmetric (Figure 1b and Figure 2(d1–d3)), medium in size: length: 32.29 (30.13–36.07) μm; width: 26.50 (24.87–30.62) μm, on average with a P/E ratio of 1.22 (Table 1).

Aperture: tricolporate. Ectoaperture: colpus, almost as long as the polar axis, open (2/3 of polar axis), straight, narrow, occasionally constricted at equator with ends acute; polar area asymmetric. Margin observed in polar view, costae colpi and fastigium conspicuous in equatorial view. Endoaperture: porus, conspicuous, lalongate, elliptic to rhomboidal in shape. Exine: tectate; exine slightly thicker in polar areas in relation to equatorial region; nexine thicker than sexine. Exine ornamentation is striate, no perforations observed. Striae relatively tight and short running parallel to the polar axis near the colpus (Figure 2(d4)).

#### 2.1.5. Morphological Characteristics of *N. tangutorum*

Pollen grains of *N. tangutorum* are perprolate in equatorial view and triangular convex to circular in polar view and monad, isopolar, and radially symmetric (Figure 1c and Figure 2(e1–e3)), medium in size: length: 42.72 (37.70–46.81) μm; width: 21.04 (19.16–23.30) μm, on average with a P/E ratio of 2.02 (Table 1).

Aperture: tricolporate. Ectoaperture: colpus, almost as long as the polar axis, open (4/5 of polar axis), straight, narrow, occasionally constricted at equator with ends acute; polar area asymmetric. Margin observed in polar view, costae colpi and fastigium conspicuous in equatorial view. Endoaperture: porus, conspicuous, lalongate, elliptic to rhomboidal in shape. Exine: tectate; exine slightly thicker in polar areas in relation to equatorial region; nexine thicker than sexine. Exine ornamentation is striate, no perforations observed (Figure 2(e4)). Striae relatively tight and short running parallel to the polar axis near the colpus while running slightly counterclockwise in the mesocolpus area, eventually forming a spiral “cluster” in one area close to a pole or running almost parallel.

#### 2.1.6. Morphological Characteristics of *N. praevisa*

Pollen grains of *N. praevisa* are perprolate in equatorial view and triangular convex to hexagonal in polar view, and they are monad, isopolar, and radially symmetric (Figure 1(d1,d2) and Figure 2(f1–f3)), medium in size: length: 37.55 (34.14–40.01) μm; width: 19.18 (16.64–21.40) μm, with an average P/E ratio of 1.96 (Table 1).

Aperture: tricolporate. Ectoaperture: colpus, almost as long as the polar axis, open (4/5 of polar axis), straight, narrow, occasionally constricted at equator with ends acute; polar area asymmetric. Margin observed in polar view, costae colpi and fastigium conspicuous in equatorial view. Endoaperture: porus, conspicuous, lalongate, elliptic to rhomboidal in shape. Exine: tectate; exine slightly thicker in polar areas in relation to equatorial region; nexine thicker than sexine. Exine ornamentation is striate, no perforations observed (Figure 2(f4)). Striae relatively tight and short running parallel to the polar axis near the colpus, while running slightly counterclockwise in the mesocolpus area, eventually forming a spiral “cluster” in one area close to a pole or running almost parallel.

## 3. Discussion

Overall, for all the analyzed taxa of Nitraria, a characteristic feature is radially symmetric, monad, isopolar pollen grains. The pollen shape is circular, pseudo-hexagonal, hexagonal, or triangular convex in polar view and perprolate to subprolate in equatorial view. The aperture is tricolporate, and colpi are long and narrow. In the comparison of average lengths of the polar and equatorial axes of *Nitraria* pollen grains, it turned out that the average length of the polar axis in *N. tangutorum*, and in most specimens of *N. schoberi* (Sariozek, Taskarasu, Karatal, Kulunda, and Malinovoe), it is greater than that in the other species, whereas the average equatorial axis length in *N. komarovii*, *N. praevisa*, and *N. sibirica* is less than that in *N. schoberi* and *N. pamirica* (Table 1). The polar axis and equatorial axis of pollen grains in the genus *Nitraria* vary continuously and overlap in their ranges. The smallest pollen grains were found in *N. komarovii* (27.15 × 16.42 μm), and the largest ones were found in specimens of *N. schoberi* (42.87 × 27.84 μm).

**Figure 3:**
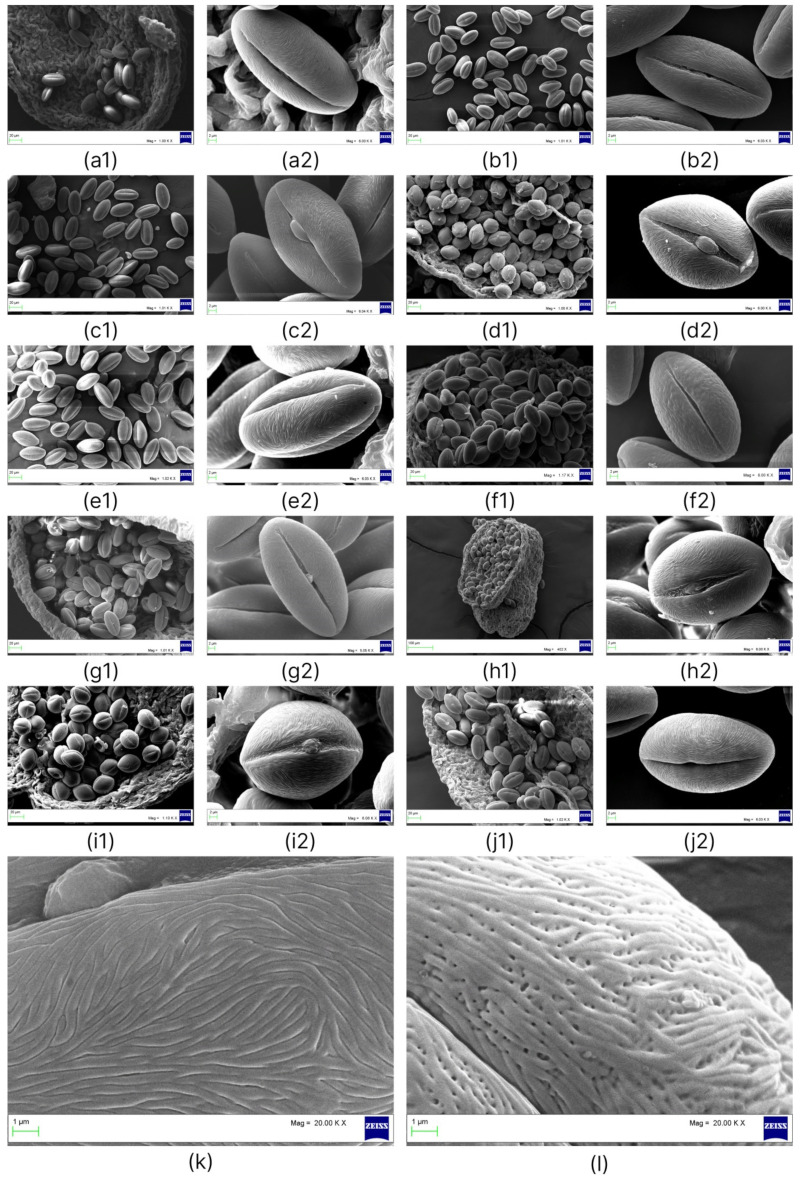
SEM micrographs of *N. sibirica* pollen. Collection sites of the photographed specimens: (**a1**,**a2**) Dauria; (**b1**,**b2**) Shelek; (**c1**,**c2**) Balansor; (**d1**,**d2**) Karatal; (**e1**,**e2**) Balhash (**f1**,**f2**) Kurti; (**g1**,**g2**) Kosh-Agach; (**h1**,**h2**) Taskarasu; (**i1**,**i2**) Basshi; (**j1**,**j2**) Koktal; (**k**) Kosh-Agach, details of the exine surface; and (**l**) Basshi, details of the exine surface.

A comparison and analysis of P/E ratios of pollen grains within the genus *Nitraria* showed that the highest P/E values belong to *N. tangutorum* and *N. praevisa* (2.02 and 1.96, respectively), and indeed, these species have a perprolate pollen shape. In *N. komarovii* and specimens of *N. schoberi*, the P/E ratio is in the range of 1.57–1.70. The lowest P/E value was detected in *N. pamirica* pollen grains (1.22), which possess a subprolate shape (Figure 2(d1–d3)). Most *N. sibirica* specimens (Balhash, Kosh-Agach, Dauria, Shelek, Balansor, Kurti, and Koktal) have P/E in the range of 1.70–1.97. By contrast, specimens of *N. sibirica* (Taskarasu, Karatal, and Basshi) have much lower P/E, 1.32–1.44, and pollen grains have a subprolate or prolate shape (Figure 3(d1,d2,h1,h2,i1,i2)).

**Figure 4:**
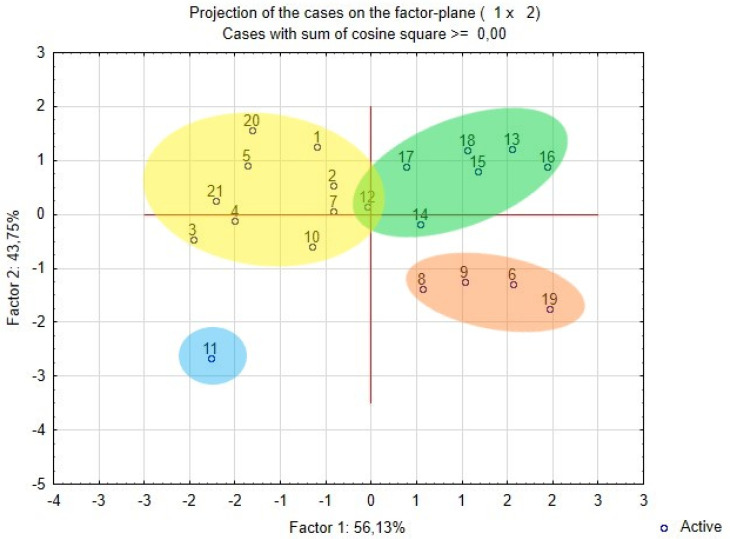
The PCA plot showing four clusters of *Nitraria* specimens. Blue ellipse: *N. komarovii*; green ellipse: *N. schoberi*; orange ellipse: *N. pamirica* (specimen No. 19) and specimens of *N. sibirica* (Basshi, Taskarasu, and Karatal); yellow ellipse: *N. tangutorum*, *N. praevisa*, and specimens of *N. sibirica* (Balhash, Kosh-Agach, Dauria, Shelek, Balansor, Kurti, and Koktal).

Principal component analysis (PCA) of the main morphological traits of pollen grains in the *Nitraria* species uncovered an obvious clustering of the specimens (Figure 4). Two principal components, PC1 and PC2, together accounted for 98.86% of the total variance. *N. komarovii* (specimen No. 11) is an outlier, whereas *N. pamirica* (specimen No. 19) and *N. sibirica* specimens Basshi, Taskarasu, and Karatal (No. 6, 8, and 9, respectively) stand out as a small group. The two remaining clusters clearly represent a group of *N. schoberi* specimens (Balhash, Sariozek, Basshi, Taskarasu, Karatal, Kulunda, and Malinovoe) and a group consisting of *N. tangutorum*, *N. praevisa*, and specimens of *N. sibirica* (Balhash, Kosh-Agach, Dauria, Shelek, Balansor, Kurti, and Koktal).

In addition, *N. sibirica* specimens Basshi, Taskarasu, and Karatal were found to have a longer equatorial axis and a shorter polar axis than do specimens of *N. sibirica* (Balhash, Kosh-Agach, Dauria, Shelek, Balansor, Kurti, and Koktal; Table 1). Differences of *N. sibirica* specimens Basshi, Taskarasu, and Karatal from the other specimens of *N. sibirica* lie in pollen grain ornamentation. In these specimens, exine ornamentation is striate and perforate. Striae are relatively loose, packed in the mesocolpia, and short (Figure 3k,l). Previously, we have documented differences between the populations of *N. sibirica* from Basshi and Taskarasu in many morphological traits of vegetative and generative organs, bush habitus [30], the profile and levels of phenolic compounds [31,32], and in the analysis of the ITS2 region of nuclear ribosomal DNA [33,34]. The above findings support the taxonomic separation of these populations.

Our results on the type and shape of pollen grains do not contradict earlier descriptions [27,28]. There are minor differences in the size of pollen grains, in the width of furrows, and in the size of pores; this can be explained by the different environmental conditions of the plant specimens in question.

**Figure 5:**
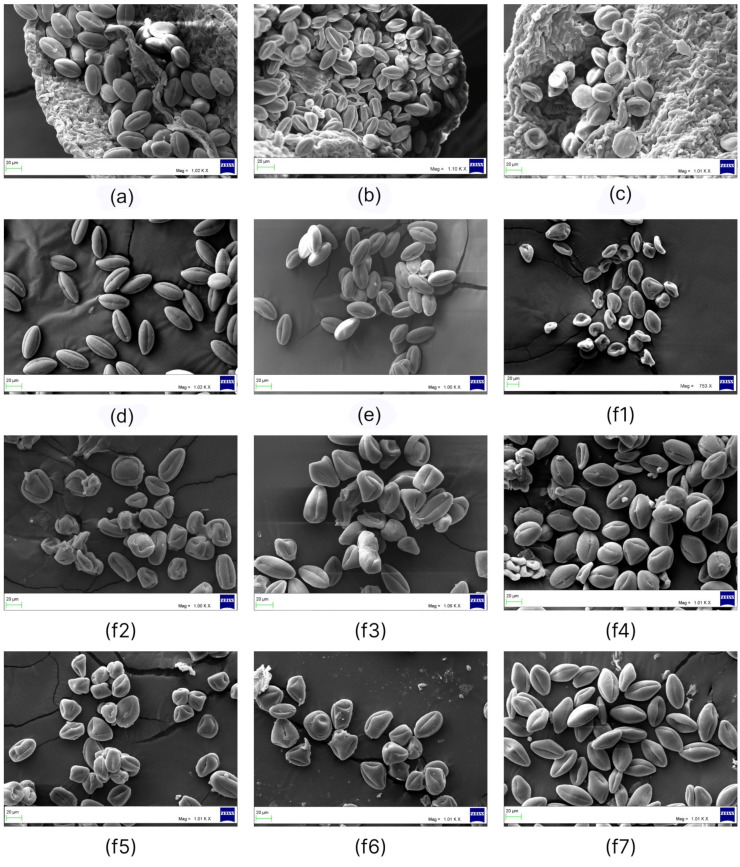
SEM micrographs of *Nitraria* pollen. (**a**) *N. sibirica*; (**b**) *N*. *komarovi*; (**c**) *N*. *pamirica*; (**d**) *N*. *tangutorum*; (**e**) *N. praevisa*; (**f1**–**f7**) *N*. *shoberi* from (**f1**) Balhash, (**f2**) Kulunda, (**f3**) Malinovoe, (**f4**) Karatal, (**f5**) Basshi, (**f6**) Taskarasu, and (**f7**) Sariozek.

When comparing pollen grains in a single anther, we noticed that in species *N. komarovii*, *N. tangutorum*, *N. praevisa*, and *N. pamirica* and in all specimens of *N. sibirica*, pollen grains have an almost stable shape and size (Figure 3(a1,b1,c1,d1,e1,f1,g1,h1,i1,j1) and Figure 5a–e). In *N. schoberi* in all populations, pollen grains of various shapes can be found (Figure 5(f1–f7)).

**Figure 6:**
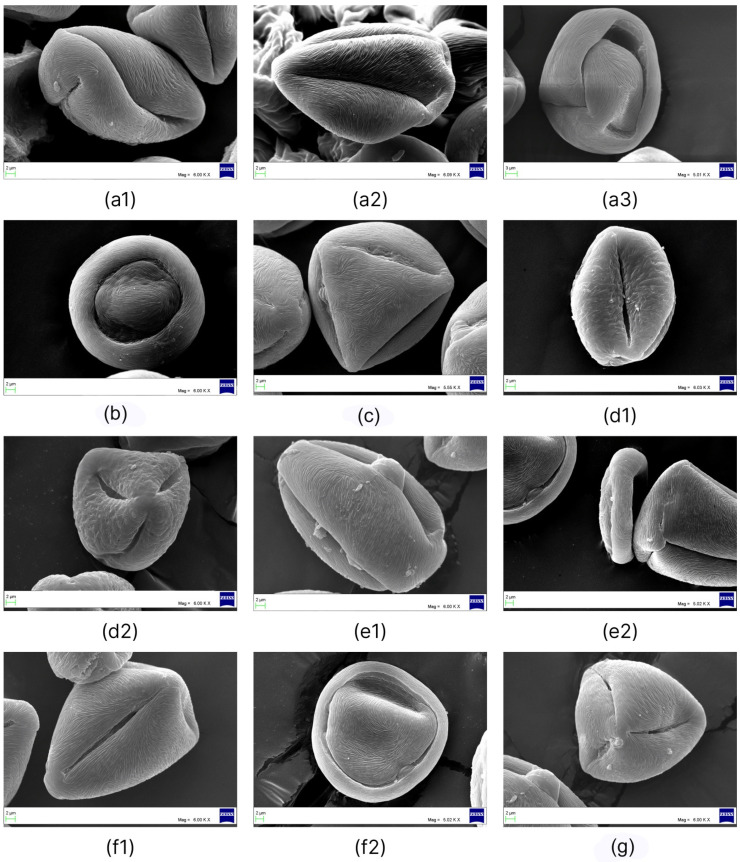
Deformed pollen grains of *N. schoberi* from (**a1**–**a3**) Kulunda, (**b**) Malinovoe, (**c**) Karatal, (**d1**,**d2**) Basshi, (**e1**,**e2**) Sariozek, (**f1**,**f2**) Taskarasu, and (**g**) Balhash.

For instance, the rounded shape occurred more often in populations Balhash, Sariozek, Karatal, Kulunda, and Malinovoe (Figure 5(f1,f2) and Figure 6(a3,b1,e2,f4)), and the conical shape occurred more often in populations Kulunda, Sariozek, and Taskarasu (Figure 6(a2,e2,f4)).

Overall, bipolar, tri-furrowed pollen grains are characteristic of *N. schoberi*. In the work of Sladkov [35], the presence of pollen grains deviating from typical ones was documented for this species. In eight populations (the Lower Volga region, the Crimea (town of Koktebel), the foothills of Dagestan, the coast of the Aral Sea, the western slope of the Mugodzhar Mountains, the southeastern shore of Lake Kainar-Kul, Kazalinsk, and Kara-Ketken), he registered four-pole four-furrowed and four-pole six-furrowed pollen grains. Along with these two types of pollen grains, that author noticed clearly deformed pollen grains with underdeveloped and irregularly oriented furrows. Agababyan and Tumanyan [27], having researched the pollen polymorphism of *N. schoberi* from various geographical locations (Semipalatinsk region (Karabelgu), Mangyshlak peninsula (Aktobe), Inner Mongolia (Bayan-Khoto), and Turchay region (Naurzum Karasu mouth)), did not find any abnormal pollen grains. An exception was a specimen of *N. schoberi* var. *caspica* Pall. from Sarepta: it had some anomalous pollen grains.

We also detected four-pole four-furrowed (Figure 6(d1,e1)) and four-pole six-furrowed (Figure 6(c1,d2,f1,g1)) pollen grains in all specimens of *N. schoberi*. The presence of such pollen grains only in populations of *N. schoberi* can serve as a diagnostic criterion for this species and requires deeper investigation into the reasons for their deformation.

## 4. Materials and Methods

### 4.1. Plant Material

**Figure 7:**
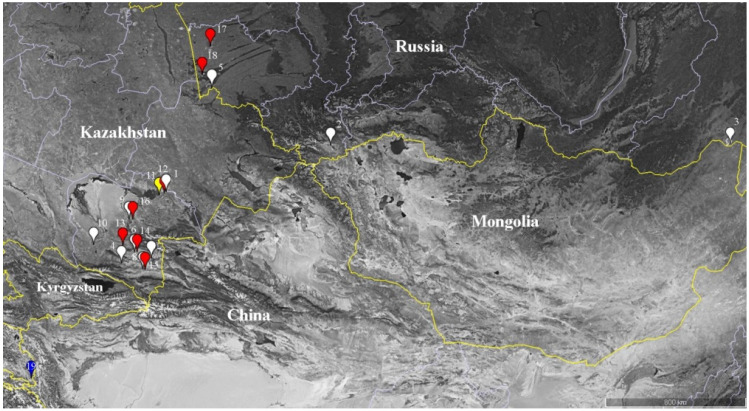
The map of sampling sites of species from the genus *Nitraria*. White color, *N. sibirica*; red color, *N. schoberi*; yellow color, *N. komarovii*; and blue color, *N. pamirica*.

We studied six species of *Nitraria* that occur in Russia (Altai Krai, Republic of Altai, Transbaikal), Kazakhstan, and Tajikistan (Figure 7). Specimens of species *N. sibirica* (10 locations), *N. schoberi* (7 locations), *N. komarovii* Iljin & Lava ex Bobrov, and *N. pamirica* L. Vassil. were collected in natural settings in 2011–2018. The collectors were E.V. Banaev and M.A. Tomoshevich. Herbarium specimens were deposited in a collection at the Central Siberian Botanical Garden, the Siberian Branch of the Russian Academy of Sciences (Herbarium of the Laboratory of Dendrology, NSK Collection, Digital Herbarium; http://herb.csbg.nsc.ru:8081 accessed on 8 September 2022). *N. tangutorum* Bobr. and *N. praevisa* were acquired as herbarium specimens that belong to original material from the Komarov Botanical Institute of the Russian Academy of Sciences (LE). The voucher information about all the specimens is shown in Table 2.

### 4.2. LM and SEM

Air-dried pollen grains of *Nitraria* species were processed by standard acetolysis [36] and were kept in glycerol. For LM, pollen grains were analyzed by means of a Carl Zeiss Axioskop 40 microscope equipped with a digital camera AxioCam MRc 5 and the AxioVision 4.8 software (Carl Zeiss, Göttingen, Germany).

For SEM examination, the air-dried pollen grains were transferred directly to a stub covered with double-sided transparent tape to prevent pollen dispersion due to pressure. The stub was inspected under a binocular microscope to ensure the pollen was distributed evenly. Pollen grains that clumped together were separated by lightly brushing with a cotton bud, and loose pollen grains were removed by a stream of dry air. The studs were coated with gold in a Mini SC 7620 sputter coater (Quorum Technologies, West Sussex, UK) and photographed under an EVO MA10 (Carl Zeiss, Germany) scanning electron microscope at 20 kV.

During this analysis, morphological traits of pollen grains were examined, including the polar axis, equatorial axis, pollen grain shape, and surface ornamentation. To be precise, the following traits were characterized: pollen size and shape (polar axis (P), equatorial axis (E), and the P/E ratio), polarity, symmetry, aperture, and exine ornamentation.

### 4.3. Data Analysis

The P and E of pollen grains were determined in 25 grains randomly chosen from 5 slides of35 each specimen by LM. All data were analyzed to calculate the mean (X), standard deviation (S), standard error (Sx), a confidence interval (95% CI), and the coefficient of variation (CV, %).

The palynological terminology is borrowed from Punt et al. [37], Hesse et al. [38], and Halbritter et al. [39]. The pollen shape class, based on the P/E ratio, was identified using Erdtman’s system [29].

Genotypes were clustered into similarity groups by PCA. All statistical analyses were performed in the STATISTICA 6.0 software (TIBCO Software Inc., Palo Alto, CA, USA).

## 5. Conclusions

In this palynomorphological study, we demonstrated that the morphological traits of pollen grains are important for the systematics of the genus *Nitraria*. Overall, for all the analyzed taxa of *Nitraria*, a characteristic feature is radially symmetric, monad, isopolar pollen grains. The pollen shape is circular, pseudo-hexagonal, hexagonal, or triangular convex in polar view and perprolate to subprolate in equatorial view. The aperture is tricolporate, and colpi are long and narrow. It was established that *N. komarovii* possesses the smallest pollen grains, and the largest ones belong to specimens of *N. schoberi*. *N. tangutorum* is characterized by the perprolate pollen shape, *N. praevisa*, *N. komarovii*, and *N. schoberi* are characterized by prolate, and *N. pamirica* is characterized by the subprolate shape. An intraspecific differentiation of *N. sibirica* was noted. In a population of *N. sibirica* (Basshi), pollen grains of a subprolate shape were detected, whereas the prolate pollen shape is characteristic of its other populations under study. Additionally, in *N. sibirica* populations Basshi, Karatal, and Taskarasu, exine ornamentation is striate–perforate, whereas the equatorial axis is longer and the polar axis is shorter as compared to specimens of *N. sibirica* (Balhash, Kosh-Agach, Dauria, Shelek, Balansor, Kurti, and Koktal). A comparison of pollen grains in one anther of *N. schoberi* in all populations revealed the presence of different shapes of pollen grains (rounded, conical, four-pole four-furrowed, and four-pole six-furrowed). The results of the current study expand the palynomorphological data for *Nitraria* and will also contribute to future phylogenetic and taxonomic studies in this genus.

## Figures and Tables

**Table 1:** Measurements (in μm) of pollen grains of *Nitraria*. The data are presented as the mean (X), standard error (Sx), and the coefficient of variation (CV, %).

No.	Taxa	Specimen	Polar Axis (P)			Equatorial Axis (E)			P/E			Shape	Ornamentation
			Range	X ± Sx	CV, %	Range	X ± Sx	CV, %	Range	X ± Sx	CV, %		
1	*N. sibirica*	Balhash	36.9–44.72	42.08 ± 0.44	5.2	20.30–24.58	22.46 ± 0.22	4.9	1.65–2.13	1.88 ± 0.02	6.5	prolate	striate
2	*N. sibirica*	Kosh-Agach	33.82–42.83	39.63 ± 0.49	6.2	20.08–24.71	22.26 ± 0.22	5.0	1.41–1.99	1.78 ± 0.02	7.3	prolate	striate
3	*N. sibirica*	Dauria	27.60–39.37	34.72 ± 0.59	8.6	13.80–21.45	18.08 ± 0.42	11.8	1.62–2.24	1.93 ± 0.03	8.9	prolate	striate
4	*N. sibirica*	Shelek	30.68–38.86	36.36 ± 0.46	6.3	15.18–21.99	19.36 ± 0.36	9.4	1.64–2.15	1.89 ± 0.02	7.2	prolate	striate
5	*N. sibirica*	Balansor	36.11–43.33	40.24 ± 0.34	4.3	17.07–23.50	20.48 ± 0.30	7.5	1.73–2.28	1.97 ± 0.02	5.8	prolate	striate
6	*N. sibirica*	Basshi	31.41–36.00	33.82 ± 0.32	4.8	21.10–27.08	25.81 ± 0.50	9.7	1.10–1.51	1.32 ± 0.02	6.7	subprolate or prolate	striate-perforate
7	*N. sibirica*	Koktal	35.92–40.28	37.90 ± 0.24	3.2	19.88–23.73	21.89 ± 0.20	4.7	1.58–1.82	1.78 ± 0.01	3.7	prolate	striate
8	*N. sibirica*	Taskarasu	28.71–36.94	33.22 ± 0.46	6.9	22.10–24.58	23.05 ± 0.13	2.9	1.28–1.65	1.44 ± 0.02	6.9	subprolate or prolate	striate-perforate
9	*N. sibirica*	Karatal	30.95–37.29	33.87 ± 0.36	5.4	23.23–26.17	24.37 ± 0.16	3.4	1.26–1.53	1.39 ± 0.01	6.1	subprolate or prolate	striate-perforate
10	*N. sibirica*	Kurti	31.95–42.72	35.33 ± 0.55	7.8	17.24–23.39	20.79 ± 0.31	7.5	1.53–1.87	1.70 ± 0.01	6.0	prolate	striate
11	*N. komarovii*	Balhash	23.38–29.50	27.15 ± 0.30	5.6	14.23–18.91	16.42 ± 0.30	9.1	1.44–1.89	1.66 ± 0.02	8.0	prolate	striate
12	*N. schoberi*	Balhash	32.59–46.32	38.33 ± 0.79	10.0	18.18–28.00	22.92 ± 0.62	13.0	1.40–2.08	1.69 ± 0.03	10.3	prolate	striate
13	*N. schoberi*	Sariozek	38.19–49.90	42.84 ± 0.62	7.2	22.28–32.59	27.84 ± 0.65	11.0	1.24–2.05	1.57 ± 0.05	16.5	prolate	striate
14	*N. schoberi*	Basshi	29.73–47.37	37.46 ± 1.03	13.0	19.32–31.78	24.07 ± 0.58	12.0	1.19–2.14	1.57 ± 0.04	13.8	prolate	striate
15	*N. schoberi*	Taskarasu	39.04–43.89	41.39 ± 0.26	3.1	24.77–27.40	26.37 ± 0.14	2.7	1.47–1.67	1.57 ± 0.01	3.1	prolate	striate
16	*N. schoberi*	Karatal	36.43–47.14	41.92 ± 0.69	8.3	23.78–31.13	28.46 ± 0.44	7.8	1.29–1.64	1.48 ± 0.01	6.4	prolate	striate
17	*N. schoberi*	Kulunda	30.66–49.06	41.17 ± 1.01	12.3	16.79–29.72	24.59 ± 0.77	15.6	1.26–2.19	1.70 ± 0.04	13.1	prolate	striate
18	*N. schoberi*	Malinovoe	35.67–50.59	42.66 ± 0.90	10.5	22.58–35.89	26.46 ± 0.71	13.4	1.30–2.16	1.63 ± 0.04	14.5	prolate	striate
19	*N. pamirica*	Shaimak	30.13–36.07	32.29 ± 0.32	4.9	24.87–30.62	26.50 ± 0.28	5.3	1.18–1.27	1.22 ± 0.005	2.0	subprolate	striate
20	*N. tangutorum*	Type	37.70–46.81	42.72 ± 0.47	5.6	19.16–23.30	21.04 ± 0.19	4.5	1.74–2.20	2.02 ± 0.02	4.8	perprolate	striate
21	*N. praevisa*	Type	34.14–40.01	37.55 ± 0.28	3.7	16.64–21.40	19.18 ± 0.23	6.2	1.75–2.37	1.96 ± 0.02	7.4	prolate or perprolate	striate

Note: see Table 2.

**Table 2:** Vaucher specimens of *Nitraria* (Nitrariaceae).

No.	Taxon	Specimen	Locality	Date	Herbarium, Specimen Number
1	*N. sibirica*	Balhash	Republic of Kazakhstan, Almaty Region, on the shore of Lake Balkhash, sandy desert	31 May2016	NSK3001248
2	*N. sibirica*	Kosh-Agach	Russia, Republic of Altay, Kosh-Agachskiy District, vicinity of Kosh-Agach village	6 July 2018	NSK3001271
3	*N. sibirica*	Dauria	Russia, Transbaikal, Borzinsky District, Daursky Reserve, on the shore of Lake Zun-Torey	8 July 2012	NSK3001266
4	*N. sibirica*	Shelek	Republic of Kazakhstan, Almaty Region, vicinity of Shelek village	26 May 2016	NSK3001242
5	*N. sibirica*	Balansor	Russia, Altai Krai, Uglovskiy District, on the shore of Lake Balansor	1 June 2011	NSK3001280
6	*N. sibirica*	Basshi	Republic of Kazakhstan, Almaty Region, vicinity of Bashshi village	25 July 2016	NSK3001249
7	*N. sibirica*	Koktal	Republic of Kazakhstan, Almaty Region, vicinity of Koktal village	25 May 2016	NSK3001236
8	*N. sibirica*	Taskarasu	Republic of Kazakhstan, Almaty Region, vicinity of Taskarasu village	26 May 2016	NSK3001244
9	*N. sibirica*	Karatal	Republic of Kazakhstan, Almaty Region, Karatalskii District, vicinity of Ushtobe city, on the terrace of the Karatal River	29 May 2016	NSK3000922
10	*N. sibirica*	Kurti	Republic of Kazakhstan, Almaty Region, Iliyskiy District, north of Kurty village, on the bank of the Kurty River	27 May 2016	NSK3001241
11	*N. komarovii*	Balhash	Republic of Kazakhstan, Almaty Region, on the shore of Lake Balkhash, sandy desert	31 May 2016	NSK3000920
12	*N. schoberi*	Balhash	Republic of Kazakhstan, Almaty Region, on the shore of Lake Balkhash, sandy desert	31 May 2016	NSK3000948
13	*N. schoberi*	Sariozek	Republic of Kazakhstan, Almaty Region, 30 km north of Saryozek village	25 May 2016	NSK3000947
14	*N. schoberi*	Basshi	Republic of Kazakhstan, Almaty Region, vicinity of Bashshi village	25 May 2016	NSK3000982
15	*N. schoberi*	Taskarasu	Republic of Kazakhstan, Almaty Region, vicinity of Taskarasu village	26 May 2016	NSK3000954
16	*N. schoberi*	Karatal	Republic of Kazakhstan, Almaty Region, Karatalskiy District, vicinity of Ushtobe town, on the terrace of the river Karatal	29 May 2016	NSK3000952
17	*N. schoberi*	Kulunda	Russia, Altai Krai, Slavgorodskiy District, on the shore of Lake Kulundinskoe	2 June 2011	NSK3000975
18	*N. schoberi*	Malinovoe	Russia, Altai Krai, Mikhailovskiy District, on the shore of Lake Malinovoe	1 June 2011	NSK3000971
19	*N. pamirica*	Shaimak	Republic of Tajikistan, Gorno-Badakhshan Autonomous Region, Eastern Pamir, on the cliff of the Djilga River	10 August 2014	NSK3001238
20	*N. tangutorum*	Type	Zaidan orientalis, in vincinity Barun-zsasak	17 June 1901	LE01025061
21	*N. praevisa*	Type	China, prov. Ganjsu 67 rm septentrionem versus ab Lanjchshou	29 June 1957	LE01043513

## Data Availability

Raw data are available upon request.
